# Climatic and Landscape Influences on Fire Regimes from 1984 to 2010 in the Western United States

**DOI:** 10.1371/journal.pone.0140839

**Published:** 2015-10-14

**Authors:** Zhihua Liu, Michael C. Wimberly

**Affiliations:** Geospatial Sciences Center of Excellence, South Dakota State University, Brookings, South Dakota, United States of America; University of California Davis, UNITED STATES

## Abstract

An improved understanding of the relative influences of climatic and landscape controls on multiple fire regime components is needed to enhance our understanding of modern fire regimes and how they will respond to future environmental change. To address this need, we analyzed the spatio-temporal patterns of fire occurrence, size, and severity of large fires (> 405 ha) in the western United States from 1984–2010. We assessed the associations of these fire regime components with environmental variables, including short-term climate anomalies, vegetation type, topography, and human influences, using boosted regression tree analysis. Results showed that large fire occurrence, size, and severity each exhibited distinctive spatial and spatio-temporal patterns, which were controlled by different sets of climate and landscape factors. Antecedent climate anomalies had the strongest influences on fire occurrence, resulting in the highest spatial synchrony. In contrast, climatic variability had weaker influences on fire size and severity and vegetation types were the most important environmental determinants of these fire regime components. Topography had moderately strong effects on both fire occurrence and severity, and human influence variables were most strongly associated with fire size. These results suggest a potential for the emergence of novel fire regimes due to the responses of fire regime components to multiple drivers at different spatial and temporal scales. Next-generation approaches for projecting future fire regimes should incorporate indirect climate effects on vegetation type changes as well as other landscape effects on multiple components of fire regimes.

## Introduction

Fire is an integral component of the earth system and plays a key role in regulating vegetation structure and ecosystem function [1–3]. Understanding the relative influences of multiple controlling factors on fire regimes is one of the fundamental objectives of fire ecology, and this knowledge is critical for improving our ability to anticipate future fire regime changes. Climatic variability is a major driver of fire in many terrestrial ecosystems, as reflected in Bradstock’s conceptual model of four climatic ‘switches’ that influence fire regimes by controlling fuel amount, fuel moisture, and fire weather at contrasting temporal scales [4]. However, fire regimes are also affected by other controls such as landscape-scale patterns of vegetation, topography, and human activities [5]. For example, recent analyses in boreal Canada found that vegetation and fuels influenced the spatial and temporal patterns of fires, even in systems where climate was considered the most limiting factor [6, 7]. Topography also influences fire regimes through its effects on fuel loads and fuel moisture via site productivity and microclimate [8]. Humans can modify fire regimes by changing ignition patterns [9] and by altering fuel amount and continuity [10]. Therefore, understanding how fire regimes respond to landscape controls in addition to climatic shifts is critical in this era of unprecedented global change, and will require research that explores the effects of multiple, interacting drivers of fire regimes [11].

**Fig 1:**
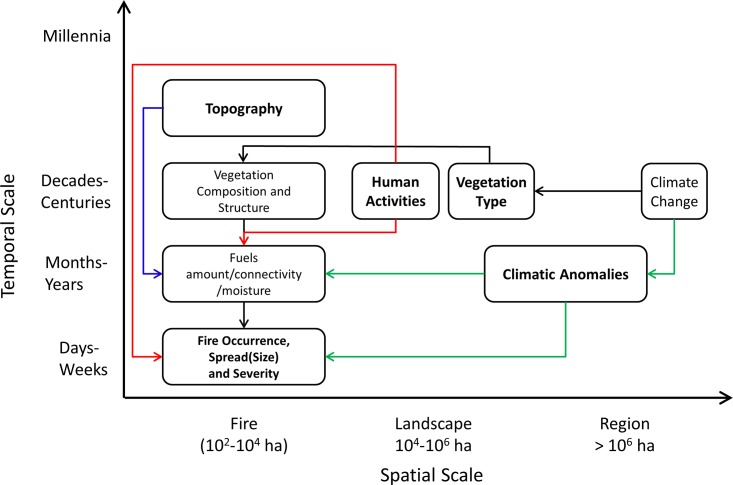
Conceptual model of major factors affecting fire occurrence, size, and severity. Red lines: human influences; green lines: direct climate influences; black lines: indirect climate (vegetation) influences; blue lines: topographic influences. Bold text represents groups of variables included in the analysis. Non-bold text represents implicit relationships that were not directly analyzed.

Fire regimes are typically described by statistical distributions of frequency, size, severity, and seasonality in a particular area during a given time period. Thus, the environmental determinants of fire regimes can be assessed by exploring how environmental drivers operating over a range of scales affect the spatial and temporal patterns of these fires (Fig 1). The behavior and effects of an individual wildfire emerge over days to weeks as a result of weather interacting with fine-grained spatial variability in fuels and vegetation. However, these interactions are also constrained by biogeographic drivers that vary over broader spatial and temporal scales. Climate, for example, is connected to fires at two distinct temporal scales [12]. Short-term climatic anomalies (months to years) affect fires by modifying vegetation growth and fuel moisture before the fire and by influencing weather during the period of fire spread. In addition, climate has more indirect, long-term (decadal or longer) effects on the distributions of major vegetation types, which in turn constrain the landscape-scale mosaics of fuels and vegetation. Topography provides a relatively stable physical template that influences fire through direct interaction with fire spread and indirect effects on vegetation, fuel amounts, and fuel moisture. Humans can affect fire through a variety of pathways including ignition, suppression, and alteration of fuels and vegetation [13]. These human impacts are in turn strongly influenced by variability in human population density, land ownership, and the resulting patterns of land use and natural resource management activities.

Because climate, vegetation, topography, and human activities interact with fire behavior and effects at different spatial and temporal scales [14, 15], they are likely to have distinctive effects on fire occurrence, size, and severity. These multiple fire regime components interact with climate along with other biophysical and human drivers to form characteristic fire regimes in different geographic settings [1]. Studies conducted at a global scale have shown that fire frequency and burned area tend to be highest in intermediate levels of productivity and moisture [16–20]. However, a more complete understanding of the other components of fire regimes, including size distribution and severity, remains rudimentary for most biomes on earth [21]. Moreover, fire size and severity have strong influences on the ecosystem structure, function, and landscape heterogeneity [22–24], and have been central to debates on whether climate change and fire management have altered fire regimes in many parts of world, including the western US [25–27]. To advance our understanding of environmental control on fire regimes, there is currently a need to explore the determinants of multiple components of the fire regime—including fire frequency, size distribution, and severity—using a comprehensive analytical framework [28].

Our overarching hypothesis in this research was that different fire regime components (occurrence, size, and severity) would exhibit characteristic spatial and spatio-temporal patterns that reflected differences in the relative importance of various environmental drivers. We used the western US as a model system to test this hypothesis because of the diversity of fire regimes spanning a broad range of vegetation and climate gradients and the availability of data on multiple characteristics of fires along with high-resolution geospatial climatic and biophysical data. We utilized an ensemble-based decision tree model to analyze how distributions of individual-fire characteristics varied continuously in space and time. We focused on large fires (> = 405 ha) because they are consistently documented across space and time, account for the majority of area burned, and have the most significant ecological and socioeconomic impacts. Specific research questions included (1) Do the spatial patterns and spatial synchrony of different fire regime components (fire occurrence, severity, and size) differ? And (2) How does the relative importance of short-term climatic anomalies, vegetation types, topography, and geographic patterns of human activity differ among these fire regime components?

## Materials and Methods

### Study Area

The study area encompassed the area west of the continental divide, which covers 2 707 515 km2 (approximately 33.5% of the conterminous US, S1 Fig). Climatic conditions vary considerably throughout the study area, ranging from desert and semi-arid regions to mountain ranges with abundant precipitation and maritime climates along the Pacific Coast. There is a high diversity of vegetation and fire regimes in the study area due to the complexity of geology, landform, and climate. For example, the coastal Pacific Northwest is dominated by temperate rainforests with infrequent, high-severity wildfires [29]. The Sierra Nevada are covered by a variety of forest, shrubland, and grassland with a mixture of different fire regimes [30]. Southern California is characterized by Mediterranean climate, and chaparral vegetation that burns at a relatively high frequency and severity [31]. The mountain ranges of the interior West are covered by a variety of forest types and associated fire regimes that vary along elevation gradients [32]. Much of the low-elevation area in the intermountain West is dominated by drought-adapted vegetation, such as shrub and grass, which historically supported a diversity of fire regimes.

### Data Sources

#### Fire dataset

Perimeter and severity data for all large wildfires (> = 405 ha) from 1984–2010 (S1 Fig) were obtained from the Monitoring Trends in Burn Severity (MTBS) project (http://www.mtbs.gov/). A total of 6071 fires (85 for wildland fire use, and 5986 for wildfire) were included in the analysis after removing duplicate records and prescribed fires. Wildland fire use (WFU) are naturally ignited wildland fires which can be managed to accomplish specific resource objectives when people are not threatened. WFU constituted 1.4% of total fires, and the inclusion or exclusion of these fires did not affect the results. The fires that are being studied are mostly those that escape initial attack, and thus likely occurred under relatively extreme fire weather conditions. Fire ignition and extinction dates were used to calculate climatic conditions before and during the fires. Fire occurrence location, size, and percent of high severity burning were used as response variables. For fire occurrence analysis, we used a case-control approach in which cases were the observed fires and controls were artificially generated fire perimeters with random locations, timing, and sizes (see Sampling Random Fire Polygons for details). Size referred to individual fire extent (mean = 3918 ha, s.d. = 11277 ha). Unburned islands within the MTBS fires are not always mapped, sometimes resulting in an overestimation of the area burned [33]. Fire severity was measured by the degree of change in vegetation (i.e., mortality, or biomass consumption) and soil (i.e., char, mineral soil, ash) one year post-fire relative to pre-fire conditions, as measured by delta normalized burn ratio (dNBR) derived from Landsat imagery [34]. Higher dNBR indicated an increase in vegetation mortality and biomass consumption, and therefore higher fire severity. The dNBR has been shown to correlate well with field-based assessments of fire severity over a broad range of ecosystem types in the western U.S. [35, 36]. Percent of high severity burning referred to the proportion of area burned by high severity fires, which corresponds to at least 80% vegetation mortality (mean = 8.83%, s.d. = 12.73%). Methods for processing and extracting the wildfire data are provided in S1 File.

### Independent Variable: Climate, Vegetation, Topography, and Human Influence

Gridded climate variables at 4 km spatial resolution, including daily maximum temperature, precipitation, wind speed, and minimum relative humidity, were downloaded from the University of Idaho (http://nimbus.cos.uidaho.edu/MACA/) [37, 38]. For each fire, daily climate variables were spatially aggregated over the grid cells whose centers were within the fire perimeters. A variety of short-term antecedent climate variables known to influence fire, summarized over 90 days preceding fire ignition [39], previous year growing seasons (May to September) [40], and previous winters (October to March) [41], were calculated as means and anomalies from climatological normals over 1981–2010. To capture post-ignition weather conditions, means and maxima were calculated for the fire spreading period, defined as the period between fire ignition and extinction dates. The mean and median lengths of the fire spreading period were 25 and 12 days respectively.

Ideally, vegetation composition and structure at the time of each fire should be used to evaluate vegetation effects on fire occurrence, size and severity. However, vegetation characteristics have changed over our 27-year study period as a result of succession, forest management, and natural disturbances, and spatially and temporally consistent vegetation information for the western US from 1984–2010 cannot be obtained from currently available national datasets. For this study, we used 30 m spatial resolution Biophysical Setting (BpS) data from LANDFIRE project (http://www.landfire.org) to characterize the broad vegetation types for each fire. BpS is generated from the current biophysical environment (climate, soils and topography) and historical fire regimes by a state-and-transition simulation approach, providing a characteristic range of structures and fuels. BpS was aggregated to generate a set of 13 vegetation types with distinctive species composition, vegetation structure, and fuels (S2 Fig & S1 Table). We overlaid this vegetation type map with fire perimeters, and computed the percent of each vegetation type within each fire perimeter.

Elevation (meter) and slope (percent) were derived from a 30 m spatial resolution digital elevation model. Aspect-related variables (e.g., heat load index and terrain shape index) were also considered, but preliminary analysis indicated that they did not exhibit significant variability among fires, and they were not used in subsequent models. A gridded river density (km/km^2^) dataset at 250 m spatial resolution was calculated from 1: 1 million scale river network data (http://nationalatlas.gov/), using the line kernel density function with bandwidth = 1000 meters in ArcGIS 10.1. The gridded topographic indices were extracted for each fire perimeter and summarized as means for each fire.

Major road network data were downloaded from the National Atlas at 1: 1 million scale (http://nationalatlas.gov/). This dataset included highways and other major transportation corridors, but not local road systems. Wildland-urban interface (WUI) data for 2000 (the approximate mid-point of our study period) were obtained from Radeloff et al [42]. Two grids at 250 m resolution were created using the Euclidean distance function in ArcGIS 10.1 to represent distance to the nearest major road and the nearest WUI. These distance surfaces were overlaid and averaged within each fire perimeter. Land ownership was obtained through Protected Areas Database of the US (http://consbio.org/products/projects/pad-us-cbi-edition), and was classified into private ownership, public (non-wilderness) ownership, and wilderness. We overlaid the land ownership map with fire perimeters, and computed the percent of each ownership type within each fire perimeter.

There were a large number of climatic and landscape variables, many of which were strongly correlated with one another and therefore redundant. We selected a subset of these variables with Pearson correlations < |0.65| according to our a priori hypotheses about the climatic drivers of fire. For the antecedent climate variables, we preferentially selected climatic anomalies for the 90 days preceding the fire and for the previous winter and growing seasons, based on the rationale that these anomalies remove the underlying spatial variability in long-term climate and emphasize temporal deviations and are therefore effective indicators of regional droughts and other meteorological fluctuations. For the period of fire spread we included mean and maximum values of climatic variables to capture the extremes, focusing on variables expected to have the strongest influences on fire behavior such as wind speed, temperature, and humidity.

### Sampling Random Fire Polygons

We used a case-control approach to evaluate the effects of landscape and climatic factors on the probability of large fire occurrence. Random control fires were sampled based on the following criteria: (1) non-burnable land cover types, including urban areas, and agriculture, were masked out based on the NLCD 2001 land cover dataset; (2) control fire sizes were sampled from the empirical distribution of fires sizes for all observed fires, and fire perimeters were assumed to be circular to remove the influence of spatial controls on fire spread; (3) the years for the control fires were sampled from a uniform distribution from 1984–2010 to remove the effect of interannual climate variability on fire occurrence; (4) the dates of control fires were sampled from the empirical distribution for all observed fires to ensure that control fires occurred within the fire season; and (5) the lengths of the periods of spread were sampled from the empirical distribution for all observed fires. The landscape and climatic variables for each random fire (controls) were processed in the same way for observed fires (cases) described above. We sampled a number of control fires equal to the total number of observed fires in the database.

## Analysis Methods

### Research Question 1: Spatial Patterns and Spatial Synchrony of Fire Regime Components

Spatial patterns of large fire occurrence were visualized by kernel smoothing [43]. We also mapped kernel-weighted mean values of total fire size and high-severity fire size (the total area of each fire that was classified as high severity). Smoothing was carried out using a 50 km radius circular window and an isotropic Gaussian kernel. Edge effects were mitigated using the border method in which points that were < 50 km from the nearest edge were excluded. A smoothed percent of high severity burning map was produced by dividing the smoothed high-severity fire size map by smoothed total fire size map. These analyses were carried out using the spatstat package [44] in R 3.0 [45]. Spearman's rank coefficient was used to assess the correlation between the spatial patterns of different fire regime components derived from kernel smoothed surfaces.

Spatial synchrony analysis was used to assess whether temporal fluctuations in fire occurrence and other fire characteristics were synchronized across large areas or more localized. We used the spline correlogram, a modification of the nonparametric covariance function (NCF), to characterize spatial synchrony. The NCF denotes how spatial covariance, an indicator of strength of spatial synchrony, varies as a function of geographic distance between locations. The spline correlogram is an adaptation of the NCF that (1) provides a direct estimate of the spatial covariance function; (2) uses a bootstrap algorithm to provide a confidence envelope for the function; and (3) can handle univariate and multivariate spatial data. We computed spline correlograms for the number of fires, mean fire size, and mean percent of high severity burning. We employed a 100 km × 100 km grid to summarize the annual number of fires, mean fire size, and mean percent of high severity burning within each grid cell for each year, and these gridded time series datasets were used to conduct the spline correlogram analyses. This resolution was selected based on a tradeoff between sample size to calculate the correlograms and the amount of data available to compute fire statistics within a grid cell. For example, larger grid sizes reduced the sample size to calculate the correlograms and increased the minimum distance between samples, while smaller grid sizes resulted in highly skewed and zero-inflated distributions of the fire statistics. The mean annual number of fires in each 100 km grid cell was 0.81 (SD = 1.64, min = 0, max = 17, CI_95%_ = [0.77, 0.85]). We use 1000 bootstrap samples to calculate confidence intervals and assumed that the NCF was isotropic. Detailed information on these techniques can be found elsewhere [46]. Spline correlogram analyses was conducted by using the ncf package [46] in R 3.0.

### Research Question 2: Relative Importance of Landscape and Climatic Controls on Fire Regime Components

We used boosted regression trees (BRT), a machine learning method, to examine the influences of landscape and climatic controls on individual fire occurrence, fire size, and percent of high severity burning. The BRT method combines the advantages of regression trees, which relate a response to their predictors by recursive binary splits, and boosting algorithms, which combine many simple models to give improved predictive performance. BRT is well-suited for ecological analyses because of its ability to handle various types of relevant variables, automatically model interactions among variables, and produce easily interpretable results [47, 48]. In BRT, trees are fitted iteratively to the residual of the existing collection of trees, which often results into higher predictive accuracy than traditional model averaging methods [48]. The final BRT model can be understood as an additive regression model in which the individual terms are simple trees.

To avoid overfitting, BRT uses learning rate (lr), tree complexity (tc), and number of trees (nt) to balance model fit and predictive performance. The lr is used to shrink the contribution of each tree as it is added to the model. The tc controls the number of nodes or variable interactions in each tree. The best combination of parameters (lr, tc and nt) that achieves maximum predictive accuracy, can be determined by 10-fold cross validation [48]. To reduce stochastic errors that might be caused by random subsampling and bagging, a bag fraction of 0.75 was used for all models; meaning that at each iteration, 75% of the data were drawn at random, without replacement, from the full training set. BRT analyses were conducted using the dismo package [48] in R 3.0.

In this analysis, we used a binomial model for large fire occurrence probability based on binary case-control fire perimeter data (observed fire perimeters-cases; random fire perimeters-controls). The best fire occurrence model was selected by maximizing the area under the receiver operating characteristic curve (0.97), with tc = 3, nt = 2600, and lr = 0.075. We used Gaussian models for fire size, and percent of high severity burning based on only the observed fires. The best fire size models were selected by maximizing the variance explained by the model (0.76), with *tc* = 3, *nt* = 1880, *lr* = 0.05. The best percent of high severity burning model was also selected by maximizing the variance explained by the model (0.60), with *tc* = 3, *nt* = 360, *lr* = 0.1.

The BRT method automatically models the interactions among variables, because every successive tree node constitutes a potential interaction. We plotted the first trees from the fire occurrence model, fire size model, and percent of high severity burning model, because displaying a single tree (usually the first tree) can provide a clear and easily interpretable depiction of complex interactions [48, 49]. We reported the variables that had a relative influence greater than 5%, along with their marginal effects on fire regime components. The relative importance of each explanatory variable was calculated by averaging over all trees in a model the number of times a variable was selected for splitting weighted by the squared residual improvement resulting from these splits. The marginal effect of a variable on fire regime components was determined from partial dependency plots, which showed the effect of a variable on the response after accounting for the average effects of all other variables [48].

We stratified the study area into forest and non-forest sub-regions based on major vegetation type of Omernik’s level III ecoregions. Non-forest subregions included ecoregions dominated by grasslands and shrublands as well as woodlands and chaparral. We repeated the analysis for each sub-region to test whether relative importance of landscape and climatic controls was consistent across the western US or differed between the two sub-regions (S3 Fig).

## Results

**Fig 2:**
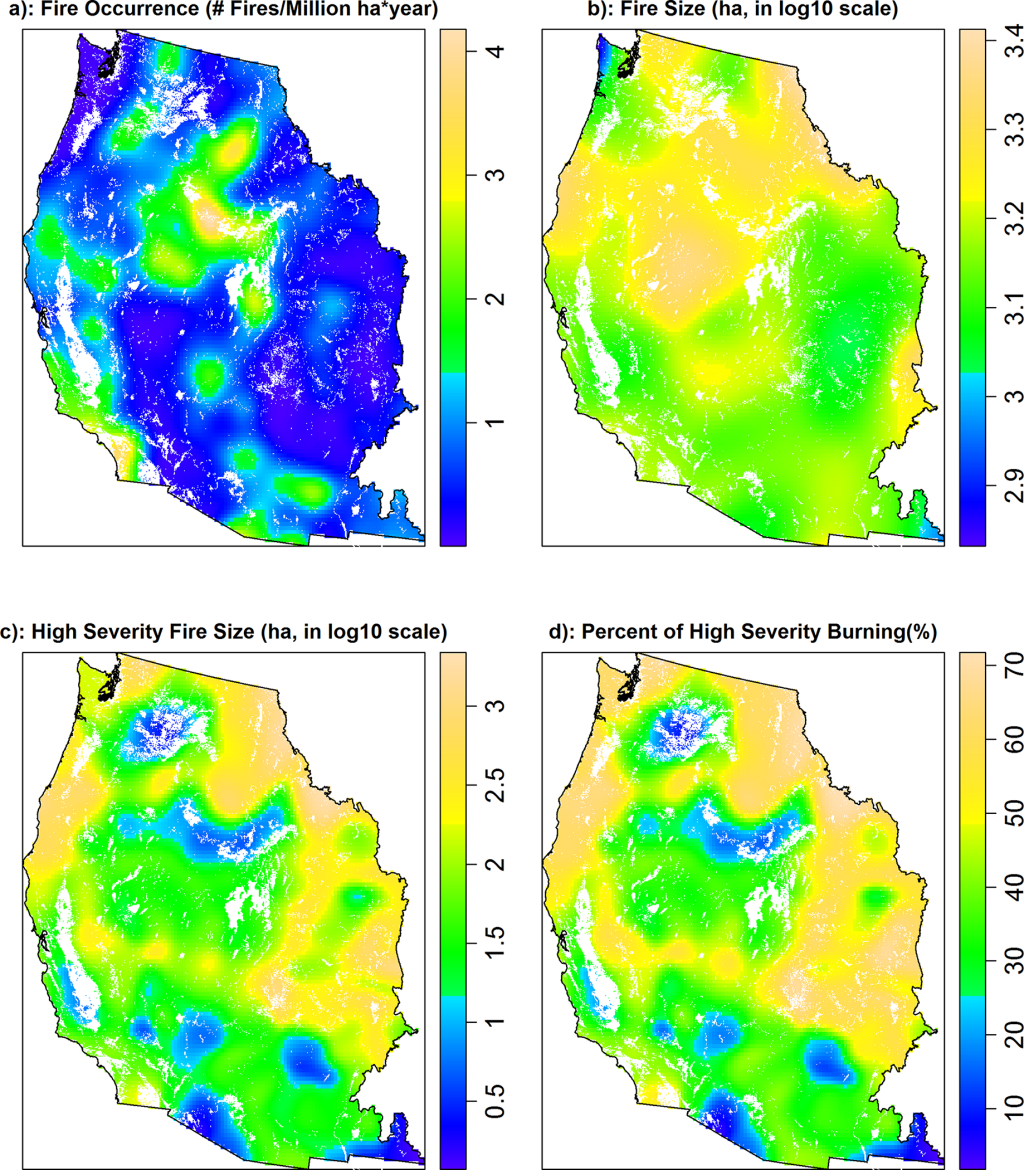
Spatial distributions of (a) smoothed density of large fires (fires per million ha per year), (b) smoothed mean fire size (ha), (c) smoothed mean high severity fire size (ha), and (d) smoothed percent of high severity burning (%) in the western US. Non-burnable areas are displayed in white.

The 6071 large fires reported in the MTBS database burned 24 265 610 ha over 1984–2010 (10.6% of the total burnable area of the western US), of which 2 979 817 ha (12.3% of the area burned and 1.3% of the total burnable area of the western US) was high severity. Fire rotations, defined as the estimated time required to burn an area of interest, were 254 and 2077 years for all fire and high severity fire, respectively, based on the total burnable area of the western US. The mean fire occurrence density was 1.37 large fires per million ha per year, and the median fire size was 1207 ha. Smoothed maps revealed that the three fire regime components were spatially heterogeneous, and their spatial patterns were not entirely concordant, suggesting they were potentially influenced by different sets of spatial controls (Fig 2a, 2b, and 2d). Spearman's rank correlation of the spatial patterns of fire regime components showed fire occurrence and size had a positive correlation (*r* = 0.34, *p*<0.001), whereas fire occurrence and percent high severity had a negative correlation (*r* = -0.30, *p*<0.001). There was a weak positive correlation between fire size and percent of high severity burning (*r* = 0.04, *p*<0.001).

**Fig 3:**
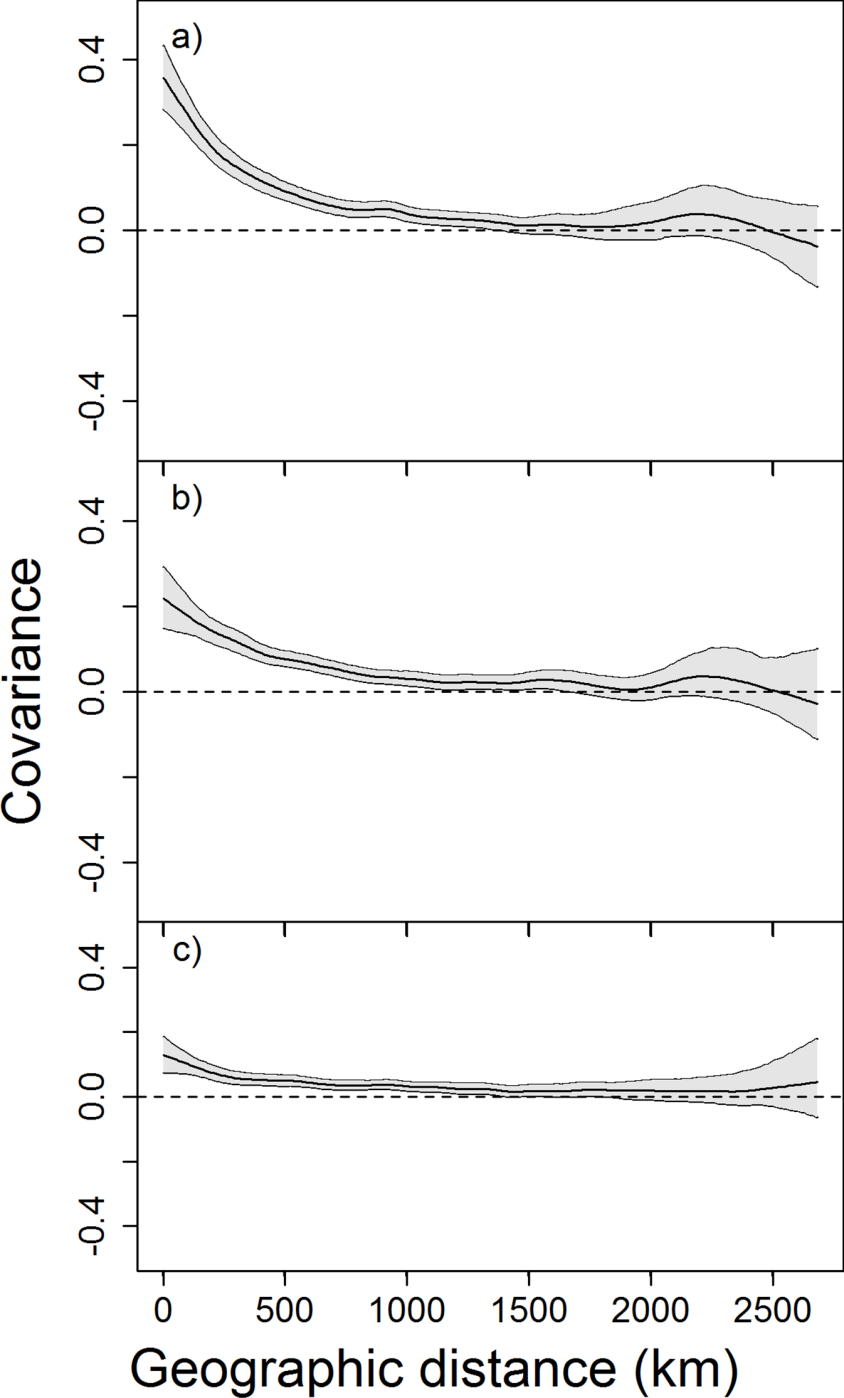
Spline correlograms illustrating the non-parametric spatial covariance function and 95% confidence intervals (gray area) for (a) large fire occurrence; (b) fire size; and (c) percent of high severity burning.

The spline correlograms showed that fire occurrence had the strongest spatial synchrony, followed by fire size, and percent of high severity burning (Fig 3). The strength of spatial covariance at the shortest lags dropped from 0.35 for fire occurrence, to 0.2 for fire size, and 0.1 for percent high severity at the grid size of 100 km. The strength of spatial covariance was stronger when larger grid sizes was used (S3 Fig), suggesting large fire tender to cluster at broader spatial scales. The values of covariance for fire regime components decreased with increasing distance, and reached zero at approximately 1500 km, which can be considered as the scale of the spatial synchrony. There was no difference of the scale of spatial synchrony among fire occurrence, size, and percent high severity. Local Indicators of Spatial Association (LISA) analysis highlighted regions with spatially synchronous fire responses and also confirmed that temporal patterns of fire occurrence tend to have stronger local associations than fire size and severity (S4 Fig).

**Fig 4:**
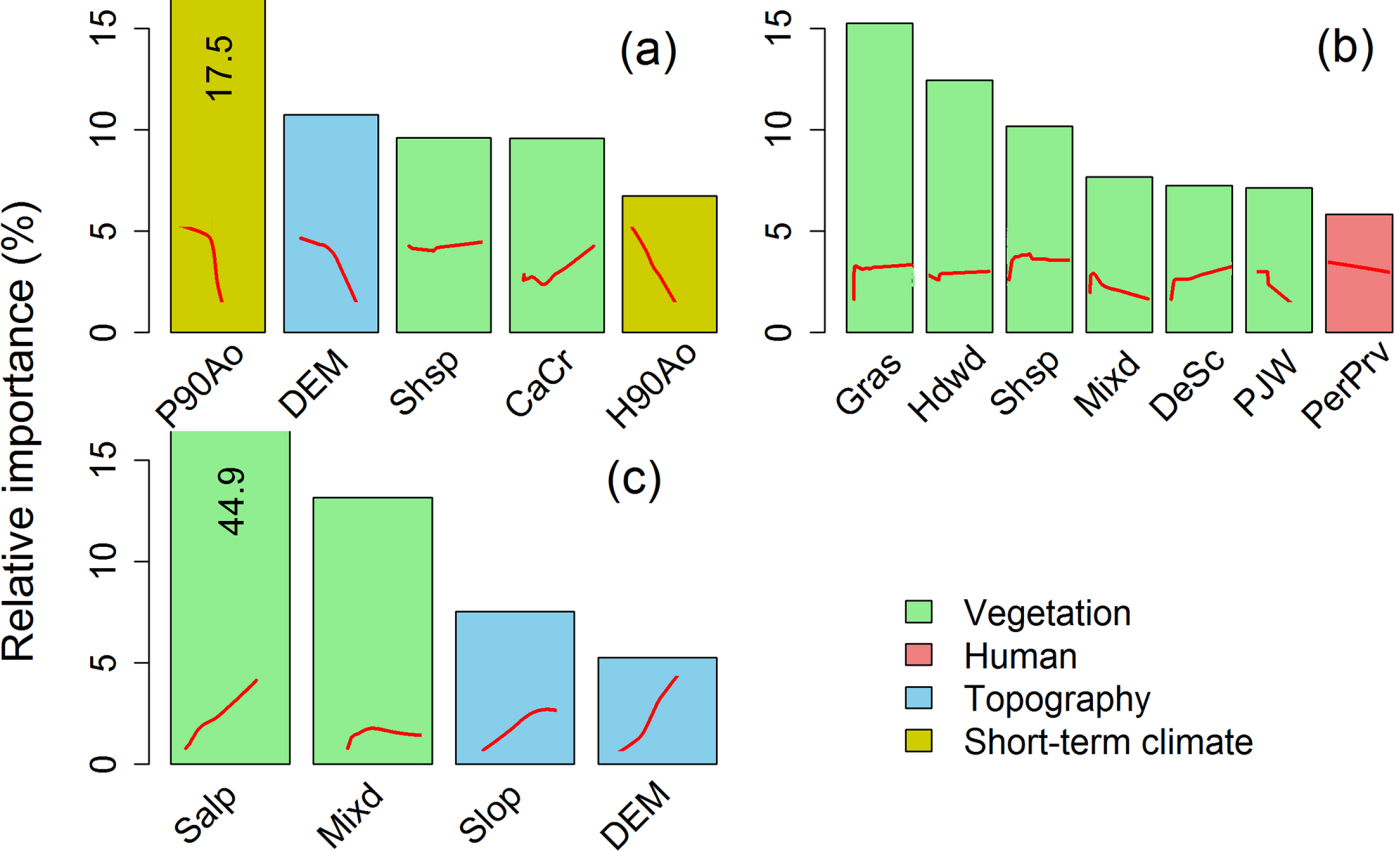
Relative influences of variables that explained greater than 5% of the variation and marginal effects (red trend lines within each bar) from boosted regression tree models for (a) large fire occurrence, (b) fire size, and (c) percent of high severity burning. Values are specified for truncated bars. Abbreviations of predictor variables and their corresponding full names are described in Table 1.

BRT results indicated that precipitation anomaly 90 days before fire ignition had the strongest influence on large fire occurrence, followed by elevation (Fig 4a). Generally, precipitation had relative stronger influences on large fire occurrence than temperature and humidity, and short-term antecedent climate conditions (90 days before fire) had a relatively stronger influence on fire occurence than long-term antecedent climate (previous winter and growing season) (S5a Fig). Elevation had a negative influence on large fire occurrence. Of the vegetation types, California Chararral and Shrub-steppe vegetation were the most influential variables, and both were positively associated with large fire occurrence (Fig 4a).

**Table 1:** Environmental variables summarized for each fire and used in the analysis.

Variable	Description	Mean+s.d
TMx (degree)	Maximum temperature during fire spread	32.36 +5.39
PMn (mm)	Mean precipitation during fire spread	0.799+1.203
HMn (%)	Mean relative humidity during fire spread	18.63+7.27
WMx (m/s)	Maximum wind speed during fire spread	3.94+1.39
WMn (m/s)	Mean wind speed during fire spread	3.24+0.80
TAo (degree)	Maximum temperature anomaly during fire spread	10.10 +5.16
PAo (mm)	Precipitation anomaly during fire spread	-0.63 +1.13
HAo (%)	Relative humidity anomaly during fire spread	-12.52+6.65
T90Ao (degree)	Mean maximum temperature anomaly for 90 days preceding fire start date	6.99 +4.55
P90Ao (mm)	Mean precipitation anomaly for 90 days preceding fire start date	-0.45 +0.90
H90Ao (%)	Relative humidity anomaly for 90 days preceding fire start date	-8.35+5.34
TPAo (degree)	Previous year growing season temperature anomaly	-0.22+1.04
PPAo (mm)	Previous year growing season precipitation anomaly	10.14+51.53
HPAo (%)	Previous year growing season relative humidity anomaly for each fire	0.19+2.86
TWAo (degree)	Previous winter temperature anomaly	8.64+1.82
PWAo (mm)	Previous winter precipitation anomaly	-0.59+0.98
TP2GAo (degree)	Growing season temperature anomaly 2 years prior	-0.078+1.28
PP2GAo (mm)	Growing season precipitation anomaly 2 years prior	-0.085+0.34
PCDF (%)	Percent Pacific coast Douglas-fir forest	0.717+6.75
InDF (%)	Percent Interior Douglas-fir	5.36+13.76
PJW (%)	Percent Pinyon-Juniper Woodland	4.81+10.87
PIPO (%)	Percent Interior Ponderosa Pine	6.17+16.25
Salp (%)	Percent Subalpine forest	6.89+19.62
Mixd (%)	Percent Mixed conifer	4.73+15.40
Hdwd (%)	Percent Hardwood	7.05+16.28
CaCr (%)	Percent California Chararral	7.84+20.26
DeSc (%)	Percent Desert scrub	10.70+24.41
MMsh (%)	Percent Mesic Mountain Shrub	1.05+4.57
Sgbr (%)	Percent Sagebrush	18.23+29.32
Shsp (%)	Percent Shrub-steppe	12.62+26.61
Gras (%)	Percent Grass	5.43+16.01
Slop (in percent)	Mean slope	14.33+10.26
DEM (m)	Mean elevation	1434+621
RiverD (km*km^-2^)	Mean river density	0.11503+0.036
D2Rd (m)	Mean distance to nearest road	11864+11240
D2WUI (m)	Mean distance to Wildland Urban interface	10432+9045
PerPrv	Percent private land	64+38
PerPub	Percent public non-wilderness land	25+34
PerWdn	Percent wilderness land	10+27

Mean and s.d. for environmental variables were calculated from all the fires.

Vegetation types were the main variables influencing fire size in the western US (Fig 4b). Of the vegetation types, Grass, Hardwood, Shrub-Steppe, Mixed Conifer, Desert scrub, Pinyon-Juniper Woodland were selected as the most influential variables. These vegetation types were positively correlated with fire size except for Mixed Conifer, Pinyon-Juniper Woodland and Hardwood forest (Fig 4b). Percent of private land was negatively associated with fire size. Topography and climate had weaker effects on fire size (S5b Fig). Human influences on fire size were stronger than many topographic and climate variables. Climate variables generally had a weak effect, and humidity during the fire spreading period and precipitation anomaly 90 days before fire ignition were the most influential climate factors (S5b Fig).

Vegetation and topographic variables had the strongest influences on percent of high severity burning (Fig 4c). Subalpine and Mixed Conifer were positively associated with percent of high severity burning. Elevation and slope were also positively associated with percent of high severity burning, likely because they were moderately (0.3–0.4) positively correlated with Subalpine, Mixed Conifer, and Pinyon-Juniper Woodland (S6 Fig). Precipitation anomaly 90 days before fire ignition was ranked as the most important climate variable influencing percent high severity, and its effect was larger than any other climate or human influence variable (S5c Fig).

**Fig 5:**
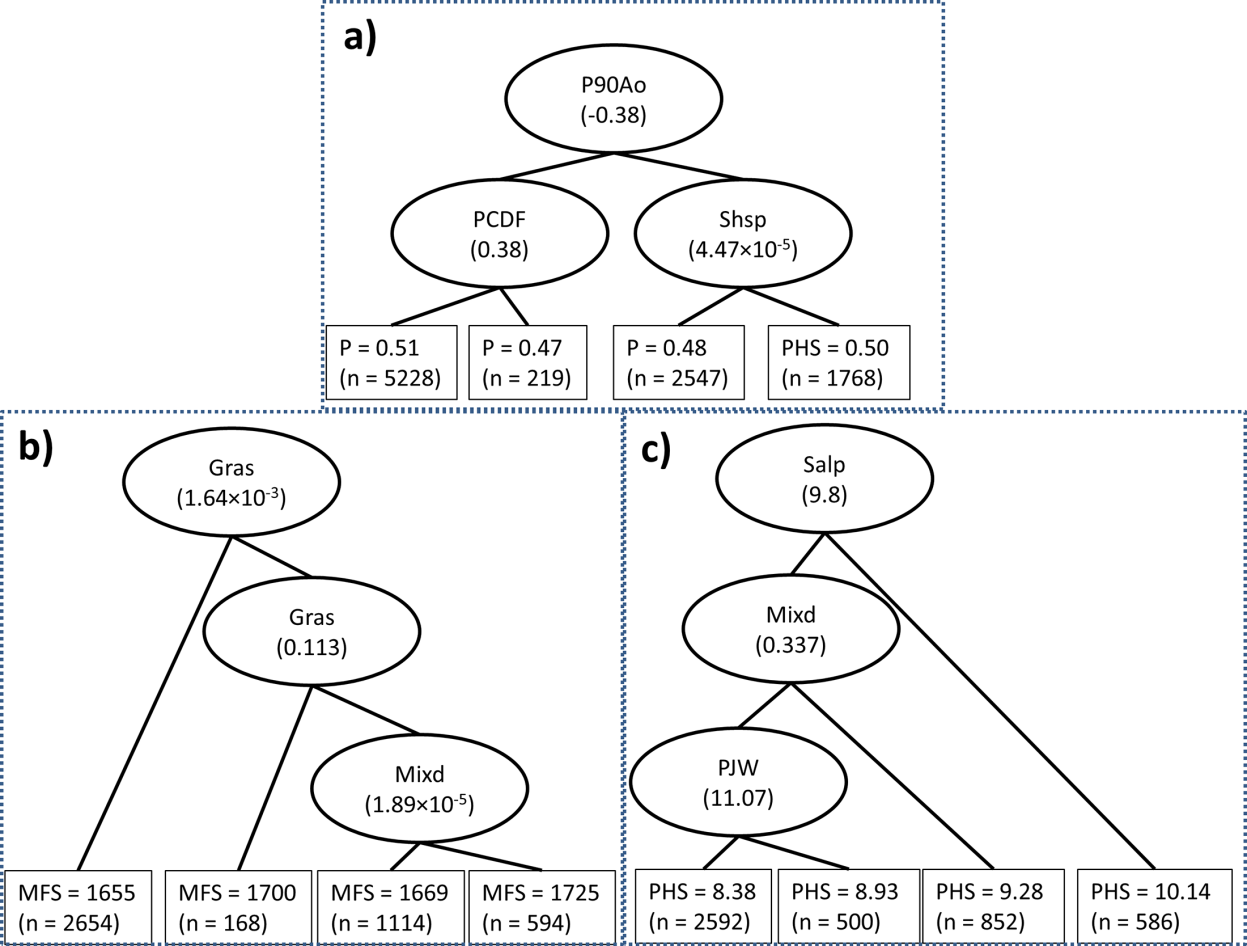
Simplified versions of the first tree of (a) fire occurrence model (b) fire size model, and (c) percent of high severity burning model computed with the boosted regression tree algorithm. The first three splits of each tree are shown to illustrate the interactions between key variables. The splitting variable and its corresponding splitting value are shown in oval above the node (variable abbreviation and units are provided in Table 1). The values in the rectangles at the terminal nodes represent the mean prediction and number of the records in the terminal nodes (n). The total number of records in the terminal nodes equals 0.75 (the bag fraction of the BRT model) of the total number of fires. Abbreviations: P: relative probability of fire occurrence; MFS: mean fire size; PHS: percent of high severity burning.

The first three nodes of the simplified BRT tree provide information about the dominant effects of climate and landscape factors on fire regime components. The first node of the fire occurrece tree showed that the precipitation anomaly 90 days before fire was the most important factor, and that it interacted with vegetation type to influence probability of large fire occurrence (Fig 5a). In contrast, the first three nodes for fire size and percent of high severity burning trees were all vegetation factors, highlighting the dominant controls of vegetation on fire size and severity (Fig 5b and 5c).

Overall, the results in forest and nonforest dominated sub-regions were similar to the results for the entire western US. Although the ranks of some individual variables differed, the relative importance of climate, vegetation, topography, and human influences were consistent for the fire regime components that we examined (S7 Fig).

## Discussion

Spatial and spatio-temporal patterns of large-fire occurrence differed from patterns of fire size and severity in the western US, and these differences can be explained by the distinctive effects of key environmental drivers on various components of the fire regime. In particular, large fire occurrence had higher spatial synchrony and was most strongly associated with short-term climatic anomalies. This finding is supported by previous analyses in the western US, which found that large fires were often preconditioned by frequent or more numerous consecutive days of hot and dry climatic conditions which result in lower fuel moisture, particularly for live fuels and larger dead fuels [39, 50, 51]. These droughts are often associated with climate patterns such as the Pacific Decadal Oscillation and El Niño Southern Oscillation, and can affect regional, inter-annual fluctuations in large fire occurrence of western North America [52, 53]. Our results are also consistent with studies employing different analytical methods based on the MTBS data in the western US. For example, Riley et al [39] found that precipitation during the past 1–3 months was a strong predictor of large fire occurrence because dead fuel moisture is strongly influenced by short-term antecedent climate conditions. Similarly, Abatzoglou and Kolden [18] found that climatic indices of drought during the current fire season had stronger relationships with area burned than antecedent climate variables from previous years, and that these regional anomalies synchronized the area burned across both forested and non-forested sub-regions of the western United States. However, our results further suggested that the short-term antecedent climatic anomalies interact with vegetation type to influence patterns of fire occurrence and have stronger influences on fire occurrence than on fire size or fire severity (Fig 5a).

Fire size and severity exhibited weaker spatial synchrony and were more strongly influenced by relatively static driving variables characterizing vegetation and topography, consistent with results from a previous study of fire severity patterns in the western US [54]. These differences reflect the fact that fire is controlled by different sets of factors at different spatio-temporal scales [55, 56], incorporating cross-scale interactions and nonlinearities at different stages of fire growth [57]. At the initiation stage, when and where a fire occurs is highly stochastic and is influenced by ignition sources and fuel moisture; the latter being largely governed by short-term antecedent climatic anomalies [58]. The initial spread of a fire involves within-patch spread stage (see Fig 1 in [57]) and depends on local fuel characteristics within the initiation patch, such as fuel type, amount, chemistry, and spatial distribution. The large fires that are being studied here all likely reached the next stage of spread among multiple patches (see Fig 1 in [57]), when fire spread and fire effects are sensitive to the spatial pattern of topography and the landscape connectivity of multiple fuel and vegetation patches. Much of the western US is topographically complex and involve sharp gradients of vegetation and fuels related to elevation, topography, and climate. Given that the large fires that we studied already tended to occur during time periods characterized by relatively dry conditions, differences in size and severity of these fires were more closely associated with vegetation and fuels than with short-term climatic variability.

Our findings of vegetation and topographic controls on fire severity are supported by a variety of other, more localized studies. For example, Steel et al [59] and Collins and Stephens [60] showed that fir-dominated mixed conifer forest in the Sierra Nevada tends to be dominated by higher fire severity than pine- or hardwood-dominated forest because of greater vertical continuity in fuels from the ground surface to the upper canopy strata. Sherriff et al [61] demonstrated that elevation and slope steepness were both positively related to high-severity fires in Colorado Front Range based on an analysis of forest structure and tree-ring fire history data. Harvey et al [62] investigated the controls on severity of five recent fires in upper-montane and subalpine forests in the U.S. Northern Rockies, and concluded that topography significantly influenced fire severity. Our regional analysis also suggested that humans had a relatively stronger influence on fire size than the other two fire regimes components, echoing previous studies that have documented the complex influences of roads and the wildland-urban interface on fire suppression and fire management policies [63, 64].

The finding that different fire regime components are controlled by different sets of drivers suggests that climate change will have varied effects on fire regimes over a range of spatial and temporal scales. Large fire occurrence in the western US was mainly influenced by short-term climate variability during the 90 days preceding the fire, which constrains fuel moisture and the availability of fine fuel to burn. Therefore, short-term variability in the frequency and duration of drought events will likely have the strongest influence on fire occurrence in western US. In contrast, fire size and severity were less affected by these climatic switches and more influenced by the spatial distribution of broad vegetation types [59, 65–67], which are more constrained by longer-term climate influences on species composition and rates of biomass accumulation and decomposition. As a result, longer-term climate-driven changes in vegetation types and associated fuel conditions may have particularly strong influences on the sizes and ecological effects of the largest fires. Future fire regime changes will thus be driven by the combined effects of both fast-changing climatic variability and slower-changing landscape factors. A subsequent study confirmed that changes in regional patterns of major vegetation types, in addition to broad scale climate, had a significant influence on future regional fire regime in the western US [68].

These results are supported by other recent studies that have documented distinctive responses of different fire regime components to environmental variability. Another study of fire regimes in the western US based on the MTBS dataset by Parks et al [69] found that burned area increased monotonically with fuel amount and had a unimodal relationship with fuel moisture, whereas fire severity increased monotonically with both fuel amount and fuel moisture. A paleoecological study of Rocky Mountain subalpine forests over the past 6000 years found that fire severity varied independently of fire frequency and was more influenced by changes in vegetation and fuels than by direct effects of climatic variation [70]. A remote sensing-based analysis found that change in vegetation and fuels due to cheatgrass (*Bromus tectorum*) invasion in recent decades has increased both fire frequency and size and has therefore substantially altered the regional fire regime across the Great Basin of the western US [71]. Simlar to the plant community reassembly that resulted from individualistic responses of tree species to historical climate change [72], the independent responses of different fire regime components to environmental change have the potential to result in novel fire regimes and produce ecological surprises in the future. These findings underscore the necessity of explicitly incorporating indirect effects of climate change on vegetation, as well as the influences of other mediating landscape variables, on multiple fire regime components in future fire projections at large spatiotemporal scales [11, 19].

Several limitations should be considered when interpreting these results. First, climate variables were measured at a much coarser spatial resolution than topography, vegetation, and human influences. However, our analysis was conducted at the fire level rather than the pixel level [54, 73, 74], and summarizing the landscape variables for each fire reduced these scale inconsistences. Second, we did not analyze human and lightning-ignited fires separately. In the future, the development of longer-term fire datasets with information on the cause of fires would be useful for exploring potential differences in human versus lightning-caused fires [9, 75]. Third, despite the widespread use of dNBR to measure fire severity in the western US, they have significant limitations including inadequate characterization of fire effects on the surface and ground layers, reduced sensitivity at higher NBR values, and inconsistent relationships with field-based measurements across different fires and vegetation types [35]. High burn severity was classified by the MTBS project using a custom threshold for each fire using based on the remote sensing indices, plot data, expert knowledge, and published literature [34]. Given the subjectivity of this procedure, we focused on the high severity class (> 80% overstory vegetation mortality), which causes the most distinctive change in spectral signatures and therefore should be more consistently classified than lower burn severity classes. However, it should be recognized that other aspects of fire severity are not be captured by this burn severity metric and that the ecological consequences of high-severity fire can vary across different environmental settings. Finally, although there was considerable interannual variability in fire activity during this 27-year period, we expect that climatic variables would be more important relative to landscape variables in analyses of longer time series that encompass a larger range of climatic conditions.

## Conclusions

In this analysis, we characterized climate and landscape effects on fire regimes across the western United States using a consistent analytical framework to produce general insights about the environmental factors that control the spatial and temporal patterns of major fire regime components. Our results showed that landscape and climatic factors had varied effects on fire occurrence, size, and severity. In particular, the spatio-temporal patterns of fire size and severity exhibited weaker spatial synchrony than fire occurrence, and were more strongly constrained by spatial patterns of vegetation, topography, and human activities. In contrast, the probability of fire occurrence was mainly influenced by recent climate anomalies, and showed a stronger spatial synchrony. These findings underscore the value of studying individualistic response of different fire regime components and ultimately incorporating multiple environmental drivers of different fire regime components into projections of future fire regimes. In particular, our results suggest the possibility that fire regimes with novel combinations of frequency, severity, and size may emerge as a result of the interacting effects of changes in climate, shifts in vegetation distributions, and continuing expansion of the human footprint. Ongoing efforts to project the effects of future global change on regional fire regimes should therefore incorporate the indirect effects of climate on vegetation type, as well as other types of landscape controls on multiple fire regime components.

## Supporting Information

S1 Fig Study area boundary overlaid by large fire locations from the MTBS dataset between 1984 and 2010 (red dots). (TIF)

S2 Fig Spatial distribution of vegetation types based on biophysical settings from the LANDFIRE project (http://www.landfire.gov/). (TIF)

S3 Fig Sensitivity of spatial covariance to different grid size (columns) for different fire regime components (rows). (TIF)

S4 Fig Local Indicators of Spatial Association (LISA) analysis for spatial synchrony of a) fire occurrence, b) fire size, and c) percent of high severity burning in the western US. Square and circle symbols indicate positive and negative associations, respectively. Sizes of the symbols indicate strength of association. Filled symbols indicate significant (p < 0.05) associations.
(TIF)

S5 Fig Relative influences of predictor variables from boosted regression tree models for (a) large fire occurrence, (b) fire size, and (c) percent of high severity burning in western US. Values are specified for truncated bars. Abbreviations of variables and their corresponding full names are described in Table 1.
(TIF)

S6 Fig Correlations among Subalpine, Mixed Conifer, Pinyon-Juniper Woodland, elevation, and slope. Abbreviations of variables and their corresponding full names are described in Table 1.
(TIF)

S7 Fig Relative influences of variables from boosted regression tree models on (a) large fire occurrence, (b) fire size, and (c) percent of high severity burning for forest dominated sub-regions, and (d) large fire occurrence, (f) fire size, and (g) percent of high severity burning for non-forest dominated sub-regions in the western US. Values are specified for truncated bars. Abbreviations of variables and their corresponding full names are described in Table 1.
(TIF)

S1 File Data source and processing procedure for fire variables. (DOCX)

S1 Table Biophysical settings reclassification used in the analysis. (CSV)

## References

[1] Bowman DMJS, Balch JK, Artaxo P, Bond WJ, Carlson JM, Cochrane MA, et al Fire in the earth system. Science. 2009;324(5926):481–4. 10.1126/science.1163886 19390038

[2] Bond W, Woodward F, Midgley G. The global distribution of ecosystems in a world without fire. New Phytol. 2005;165(2):525–38. 15720663 10.1111/j.1469-8137.2004.01252.x

[3] Pausas JG, Keeley JE. A burning story: the role of fire in the history of life. BioScience. 2009;59(7):593–601.

[4] Bradstock RA. A biogeographic model of fire regimes in Australia: current and future implications. Global Ecol Biogeogr. 2010;19(2):145–58.

[5] Bowman D, Balch J, Artaxo P, Bond WJ, Cochrane MA, D'Antonio CM, et al The human dimension of fire regimes on Earth. J Biogeogr. 2011;38(12):2223–36. 22279247 10.1111/j.1365-2699.2011.02595.x PMC3263421

[6] Parisien M-A, Parks SA, Krawchuk MA, Little JM, Flannigan MD, Gowman LM, et al An analysis of controls on fire activity in boreal Canada: comparing models built with different temporal resolutions. Ecol Appl. 2014;24(6):1341–56. 29160658 10.1890/13-1477.1

[7] Heon J, Arseneault D, Parisien MA. Resistance of the boreal forest to high burn rates. Proc Natl Acad Sci U S A. 2014;111(38):13888–93. 10.1073/pnas.1409316111 25201981 PMC4183332

[8] Rollins MG, Morgan P, Swetnam T. Landscape-scale controls over 20th century fire occurrence in two large Rocky Mountain (USA) wilderness areas. Landscape Ecol. 2002;17(6):539–57.

[9] Liu Z, Yang J, Chang Y, Weisberg PJ, He HS. Spatial patterns and drivers of fire occurrence and its future trend under climate change in a boreal forest of Northeast China. Global Change Biol. 2012;18(6):2041–56.

[10] Pausas JG, Fernández-Muñoz S. Fire regime changes in the Western Mediterranean Basin: from fuel-limited to drought-driven fire regime. Clim Change. 2012;110(1–2):215–26.

[11] Bowman DMJS, Murphy BP, Williamson GJ, Cochrane MA. Pyrogeographic models, feedbacks and the future of global fire regimes. Global Ecol Biogeogr. 2014;23(7):821–4.

[12] Hessl AE. Pathways for climate change effects on fire: Models, data, and uncertainties. Prog Phys Geogr. 2011;35(3):393–407.

[13] Liu Z, Wimberly M, Lamsal A, Sohl T, Hawbaker T. Climate change and wildfire risk in an expanding wildland—urban interface: a case study from the Colorado Front Range Corridor. Landscape Ecol. 2015: 10.1007/s10980-015-0222-4

[14] Falk DA, Heyerdahl EK, Brown PM, Farris C, Fulé PZ, McKenzie D, et al Multi-scale controls of historical forest-fire regimes: new insights from fire-scar networks. Front Ecol Environ. 2011;9(8):446–54.

[15] Liu Z, Yang J, He HS. Identifying the Threshold of Dominant Controls on Fire Spread in a Boreal Forest Landscape of Northeast China. PLoS ONE. 2013;8(1):e55618 10.1371/journal.pone.0055618 23383247 PMC3561322

[16] Archibald S, Roy DP, van Wilgen BW, Scholes RJ. What limits fire? An examination of drivers of burnt area in Southern Africa. Global Change Biol. 2009;15(3):613–30.

[17] Krawchuk M, Moritz M. Constraints on global fire activity vary across a resource gradient. Ecology. 2011;92(1):121–32. 21560682 10.1890/09-1843.1

[18] Abatzoglou JT, Kolden CA. Relationships between climate and macroscale area burned in the western United States. Int J Wildland Fire. 2013;22(7):1003–20.

[19] Morton D, Collatz G, Wang D, Randerson J, Giglio L, Chen Y. Satellite-based assessment of climate controls on US burned area. Biogeosciences. 2013;10:247–60.

[20] Pausas JG, Ribeiro E. The global fire—productivity relationship. Global Ecol Biogeogr. 2013;22(6):728–36.

[21] Bowman DM, O'Brien JA, Goldammer JG. Pyrogeography and the global quest for sustainable fire management. Annual Review of Environment and Resources. 2013;38(1):57–80.

[22] Yates CP, Edwards AC, Russell-Smith J. Big fires and their ecological impacts in Australian savannas: size and frequency matters. Int J Wildland Fire. 2008;17(6):768–81.

[23] Turner MG. Disturbance and landscape dynamics in a changing world. Ecology. 2010;91(10):2833–49. 21058545 10.1890/10-0097.1

[24] Liu Z, Yang J. Quantifying ecological drivers of ecosystem productivity of the early-successional boreal Larix gmelinii forest. Ecosphere. 2014;5(7):84 10.1890/ES13-00372.1

[25] Stephens S, Agee J, Fulé P, North M, Romme W, Swetnam T, et al Managing forests and fire in changing climates. Science. 2013;342(6154):41–2. 10.1126/science.1240294 24092714

[26] Williams MA, Baker WL. Spatially extensive reconstructions show variable-severity fire and heterogeneous structure in historical western United States dry forests. Global Ecol Biogeogr. 2012;21(10):1042–52.

[27] Odion DC, Hanson CT, Arsenault A, Baker WL, DellaSala DA, Hutto RL, et al Examining historical and current mixed-severity fire regimes in ponderosa pine and mixed-conifer forests of western North America. PloS one. 2014;9(2):e87852 10.1371/journal.pone.0087852 24498383 PMC3912150

[28] Krawchuk MA, Moritz MA. Burning issues: statistical analyses of global fire data to inform assessments of environmental change. Environmetrics. 2014;25(6):472–81.

[29] Wimberly MC, Liu Z. Interactions of climate, fire, and management in future forests of the Pacific Northwest. For Ecol Manage. 2014;327(0):270–9.

[30] Miller J, Collins B, Lutz J, Stephens S, van Wagtendonk J, Yasuda D. Differences in wildfires among ecoregions and land management agencies in the Sierra Nevada region, California, USA. Ecosphere 3 (9): 80 2012.

[31] Jin Y, Randerson JT, Faivre N, Capps S, Hall A, Goulden ML. Contrasting controls on wildland fires in Southern California during periods with and without Santa Ana winds. Journal of Geophysical Research: Biogeosciences. 2014:2013JG002541.

[32] Noss RF, Franklin JF, Baker WL, Schoennagel T, Moyle PB. Managing fire-prone forests in the western United States. Front Ecol Environ. 2006;4(9):481–7.

[33] Sparks AM, Boschetti L, Smith AMS, Tinkham WT, Lannom KO, Newingham BA. An accuracy assessment of the MTBS burned area product for shrub—steppe fires in the northern Great Basin, United States. Int J Wildland Fire. 2015;24(1):70–8.

[34] Eidenshink J, Schwind B, Brewer K, Zhu Z-L, Quayle B, Howard S. A project for monitoring trends in burn severity. Fire Ecol. 2007;3(1):1–19.

[35] French NH, Kasischke ES, Hall RJ, Murphy KA, Verbyla DL, Hoy EE, et al Using Landsat data to assess fire and burn severity in the North American boreal forest region: an overview and summary of results. Int J Wildland Fire. 2008;17(4):443–62.

[36] Miller JD, Thode AE. Quantifying burn severity in a heterogeneous landscape with a relative version of the delta Normalized Burn Ratio (dNBR). Remote Sens Environ. 2007;109(1):66–80.

[37] Abatzoglou JT. Development of gridded surface meteorological data for ecological applications and modelling. Int J Climatology. 2013;33(1):121–31.

[38] Abatzoglou JT, Brown TJ. A comparison of statistical downscaling methods suited for wildfire applications. Int J Climatology. 2012;32(5):772–80.

[39] Riley KL, Abatzoglou JT, Grenfell IC, Klene AE, Heinsch FA. The relationship of large fire occurrence with drought and fire danger indices in the western USA, 1984–2008: the role of temporal scale. Int J Wildland Fire. 2013;22(7):894–909.

[40] Littell JS, McKenzie D, Peterson DL, Westerling AL. Climate and wildfire area burned in western US ecoprovinces, 1916–2003. Ecol Appl. 2009;19(4):1003–21. 19544740 10.1890/07-1183.1

[41] Westerling AL, Hidalgo HG, Cayan DR, Swetnam TW. Warming and earlier spring increase western US forest wildfire activity. Science. 2006;313(5789):940–3. 16825536 10.1126/science.1128834

[42] Radeloff VC, Hammer RB, Stewart SI, Fried JS, Holcomb SS, McKeefry JF. The wildland-urban interface in the United States. Ecol Appl. 2005;15(3):799–805.

[43] Diggle PJ. Statistical analysis of spatial point patterns, 2nd edition: Oxford University Press, New York; 2003.

[44] Baddeley A, Turner R. Spatstat: an R package for analyzing spatial point patterns. J Stat Soft. 2005;12(6):1–42.

[45] R Core Team. R: A language and environment for statistical computing. R Foundation for Statistical Computing, Vienna, Austria. URL http://www.R-project.org/. 2013.

[46] Bjørnstad ON, Ims RA, Lambin X. Spatial population dynamics: analyzing patterns and processes of population synchrony. Trends Ecol Evol. 1999;14(11):427–32. 10511718 10.1016/s0169-5347(99)01677-8

[47] De'Ath G. Boosted trees for ecological modeling and prediction. Ecology. 2007;88(1):243–51. 17489472 10.1890/0012-9658(2007)88[243:btfema]2.0.co;2

[48] Elith J, Leathwick J, Hastie T. A working guide to boosted regression trees. J Anim Ecol. 2008;77(4):802–13. 10.1111/j.1365-2656.2008.01390.x 18397250

[49] Parisien M-A, Moritz MA. Environmental controls on the distribution of wildfire at multiple spatial scales. Ecol Monogr. 2009;79(1):127–54.

[50] Meyn A, White PS, Buhk C, Jentsch A. Environmental drivers of large, infrequent wildfires: the emerging conceptual model. Prog Phys Geogr. 2007;31(3):287–312.

[51] Schoennagel T, Veblen TT, Romme WH. The interaction of fire, fuels, and climate across Rocky Mountain forests. Bioscience. 2004;54(7):661–76.

[52] Kitzberger T, Brown PM, Heyerdahl EK, Swetnam TW, Veblen TT. Contingent Pacific—Atlantic Ocean influence on multicentury wildfire synchrony over western North America. PNAS. 2007;104(2):543–8. 17197425 10.1073/pnas.0606078104 PMC1766421

[53] Swetnam TW, Betancourt JL. Fire-southern oscillation relations in the southwestern United States. Science. 1990;249:1017–20. 17789609 10.1126/science.249.4972.1017

[54] Dillon GK, Holden ZA, Morgan P, Crimmins MA, Heyerdahl EK, Luce CH. Both topography and climate affected forest and woodland burn severity in two regions of the western US, 1984 to 2006. Ecosphere. 2011;2(12):130 10.1890/ES11-00271.1

[55] Pyne SJ, Andrews PL, Laven RD. Introduction to wildland fire: John Wiley and Sons; 1996.

[56] Moritz MA, Morais ME, Summerell LA, Carlson J, Doyle J. Wildfires, complexity, and highly optimized tolerance. Proceedings of the National Academy of Sciences of the United States of America. 2005;102(50):17912 16332964 10.1073/pnas.0508985102 PMC1312407

[57] Peters DPC, Pielke RA Sr, Bestelmeyer BT, Allen CD, Munson-McGee S, Havstad KM. Cross-scale interactions, nonlinearities, and forecasting catastrophic events. PNAS. 2004;101(42):15130–5. 15469919 10.1073/pnas.0403822101 PMC523446

[58] Pyke DA, Brooks ML, D'Antonio C. Fire as a restoration tool: a decision framework for predicting the control or enhancement of plants using fire. Restor Ecol. 2010;18(3):274–84.

[59] Steel ZL, Safford HD, Viers JH. The fire frequency-severity relationship and the legacy of fire suppression in California forests. Ecosphere. 2015;6(1):art8.

[60] Collins BM, Stephens SL. Stand-replacing patches within a ‘mixed severity’fire regime: quantitative characterization using recent fires in a long-established natural fire area. Landscape Ecol. 2010;25(6):927–39.

[61] Sherriff RL, Platt RV, Veblen TT, Schoennagel TL, Gartner MH. Historical, observed, and modeled wildfire severity in montane forests of the Colorado Front Range. PloS one. 2014;9(9):e106971 10.1371/journal.pone.0106971 25251103 PMC4175072

[62] Harvey BJ, Donato DC, Turner MG. Recent mountain pine beetle outbreaks, wildfire severity, and postfire tree regeneration in the US Northern Rockies. PNAS. 2014;111(42):15120–5. 10.1073/pnas.1411346111 25267633 PMC4210318

[63] Syphard AD, Radeloff VC, Keeley JE, Hawbaker TJ, Clayton MK, Stewart SI, et al Human influence on california fire regimes. Ecol Appl. 2007;17(5):1388–402. 17708216 10.1890/06-1128.1

[64] Haire SL, McGarigal K, Miller C. Wilderness shapes contemporary fire size distributions across landscapes of the western United States. Ecosphere. 2013;4(1):art15 http://dx.doi.org/0.1890/ES12-00257.1.

[65] Miller J, Skinner C, Safford H, Knapp EE, Ramirez C. Trends and causes of severity, size, and number of fires in northwestern California, USA. Ecol Appl. 2012;22(1):184–203. 22471083 10.1890/10-2108.1

[66] Miller J, Safford H, Crimmins M, Thode A. Quantitative evidence for increasing forest fire severity in the Sierra Nevada and southern Cascade Mountains, California and Nevada, USA. Ecosystems. 2009;12(1):16–32.

[67] Mallek C, Safford H, Viers J, Miller J. Modern departures in fire severity and area vary by forest type, Sierra Nevada and southern Cascades, California, USA. Ecosphere. 2013;4(12):art153.

[68] Liu Z, Wimberly MC. Direct and indirect effects of climate change on projected future fire regimes in the western United States. Sci Total Environ. In review(0). 10.1016/j.scitotenv.2015.10.093 26519568

[69] Parks SA, Parisien M-A, Miller C, Dobrowski SZ. Fire activity and severity in the western US vary along proxy gradients representing fuel amount and fuel moisture. PLoS ONE. 2014;9(6):e99699 10.1371/journal.pone.0099699 24941290 PMC4062429

[70] Higuera PE, Briles CE, Whitlock C. Fire-regime complacency and sensitivity to centennial-through millennial-scale climate change in Rocky Mountain subalpine forests, Colorado, USA. J Ecol. 2014;102(6):1429–41.

[71] Balch JK, Bradley BA, D'Antonio CM, Gómez-Dans J. Introduced annual grass increases regional fire activity across the arid western USA (1980–2009). Global Change Biol. 2013;19(1):173–83. 10.1111/gcb.12046 23504729

[72] Davis MB, Shaw RG. Range shifts and adaptive responses to Quaternary climate change. Science. 2001;292(5517):673–9. 11326089 10.1126/science.292.5517.673

[73] Collins BM, Kelly M, van Wagtendonk JW, Stephens SL. Spatial patterns of large natural fires in Sierra Nevada wilderness areas. Landscape Ecol. 2007;22(4):545–57.

[74] Holden ZA, Morgan P, Evans JS. A predictive model of burn severity based on 20-year satellite-inferred burn severity data in a large southwestern US wilderness area. For Ecol Manage. 2009;258(11):2399–406.

[75] Narayanaraj G, Wimberly MC. Influences of forest roads on the spatial patterns of human-and lightning-caused wildfire ignitions. Appl Geo. 2012;32(2):878–88.

