# Supplementary figures and images for: Climatic and Landscape Influences on Fire Regimes from 1984 to 2010 in the Western United States
Source: PLoS One. 2015 Oct 14;10(10):e0140839. doi: 10.1371/journal.pone.0140839 (PMC4605733; doi:10.1371/journal.pone.0140839)

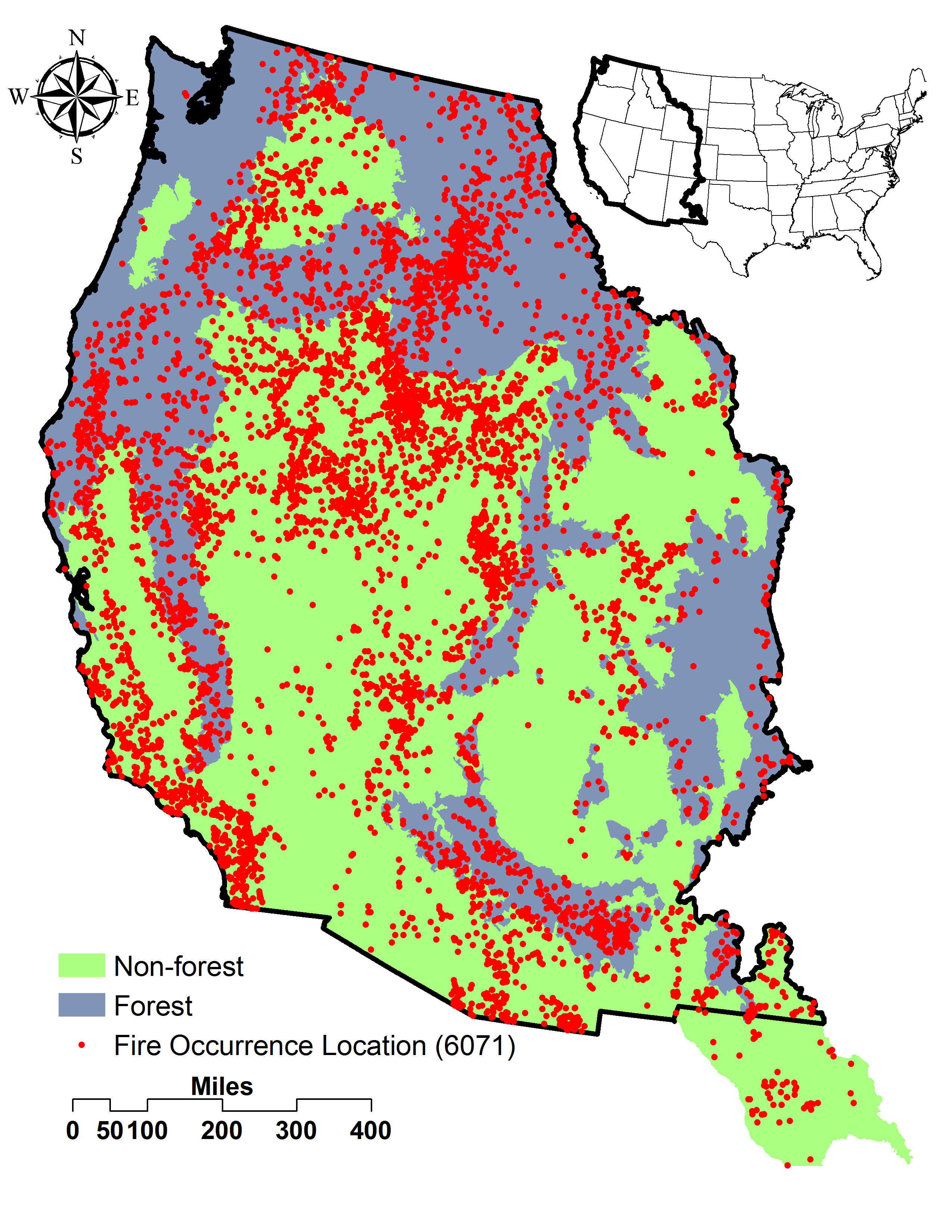

Supplement: S1 Fig — (TIF) [file pone.0140839.s001.tif]

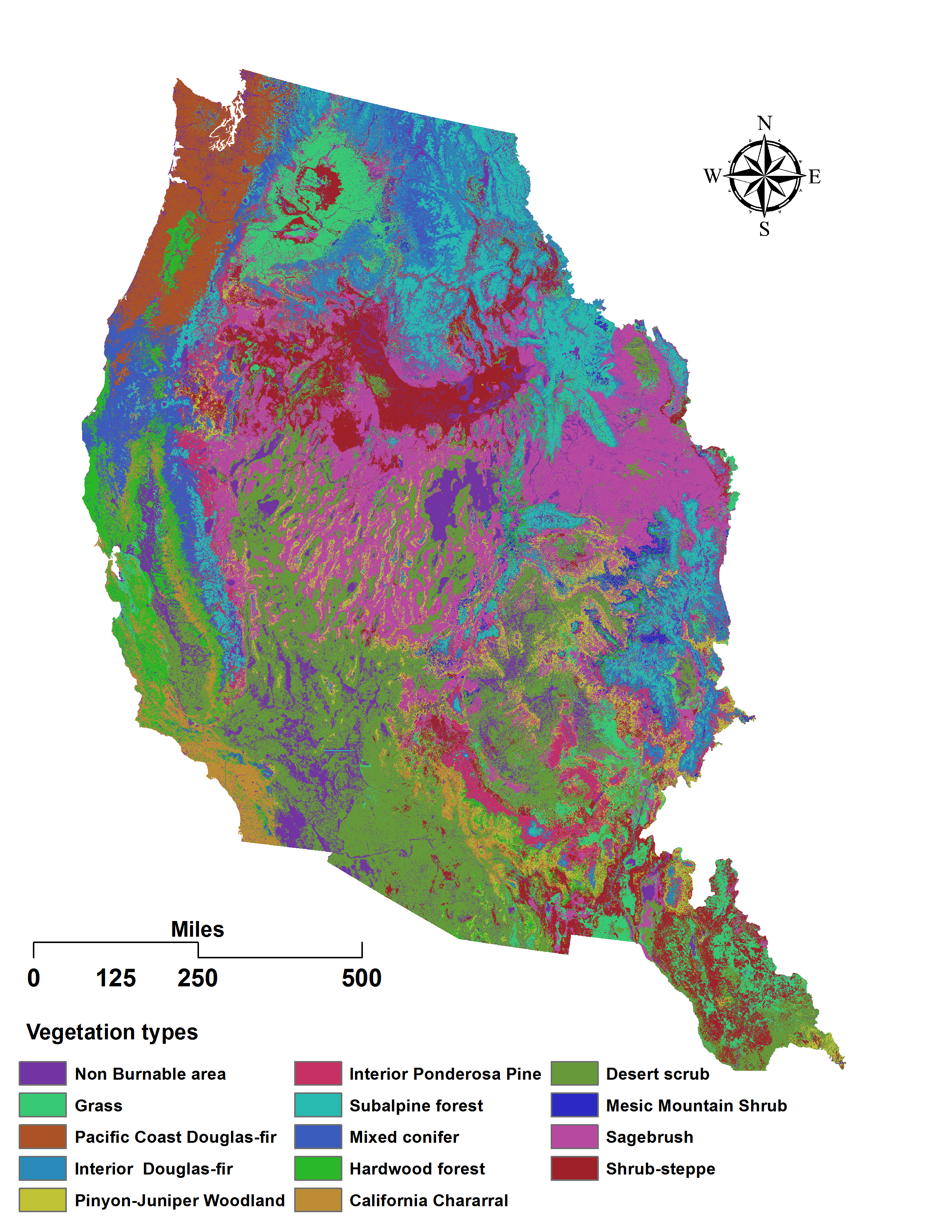

Supplement: S2 Fig — (TIF) [file pone.0140839.s002.tif]

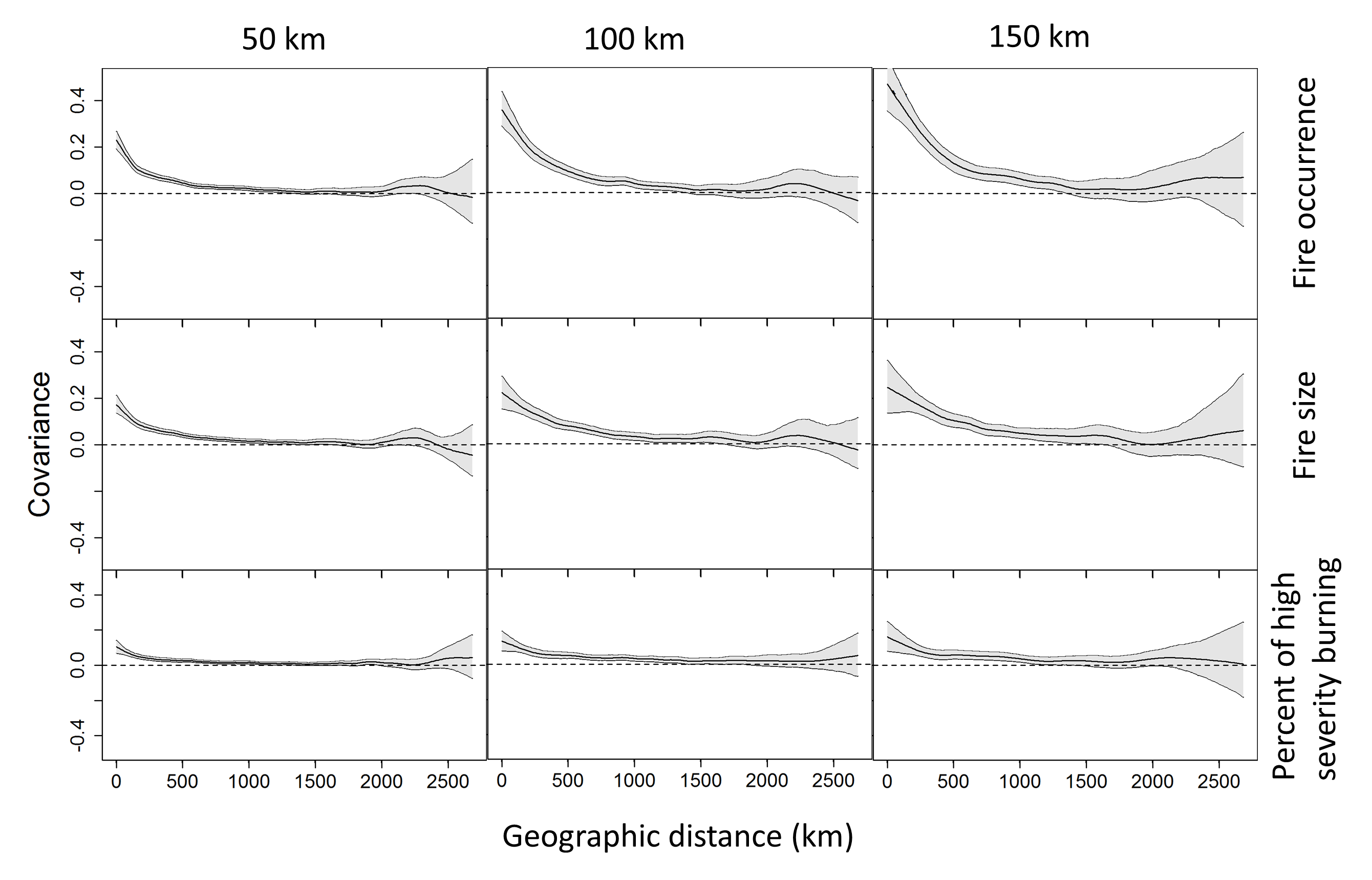

Supplement: S3 Fig — (TIF) [file pone.0140839.s003.tif]

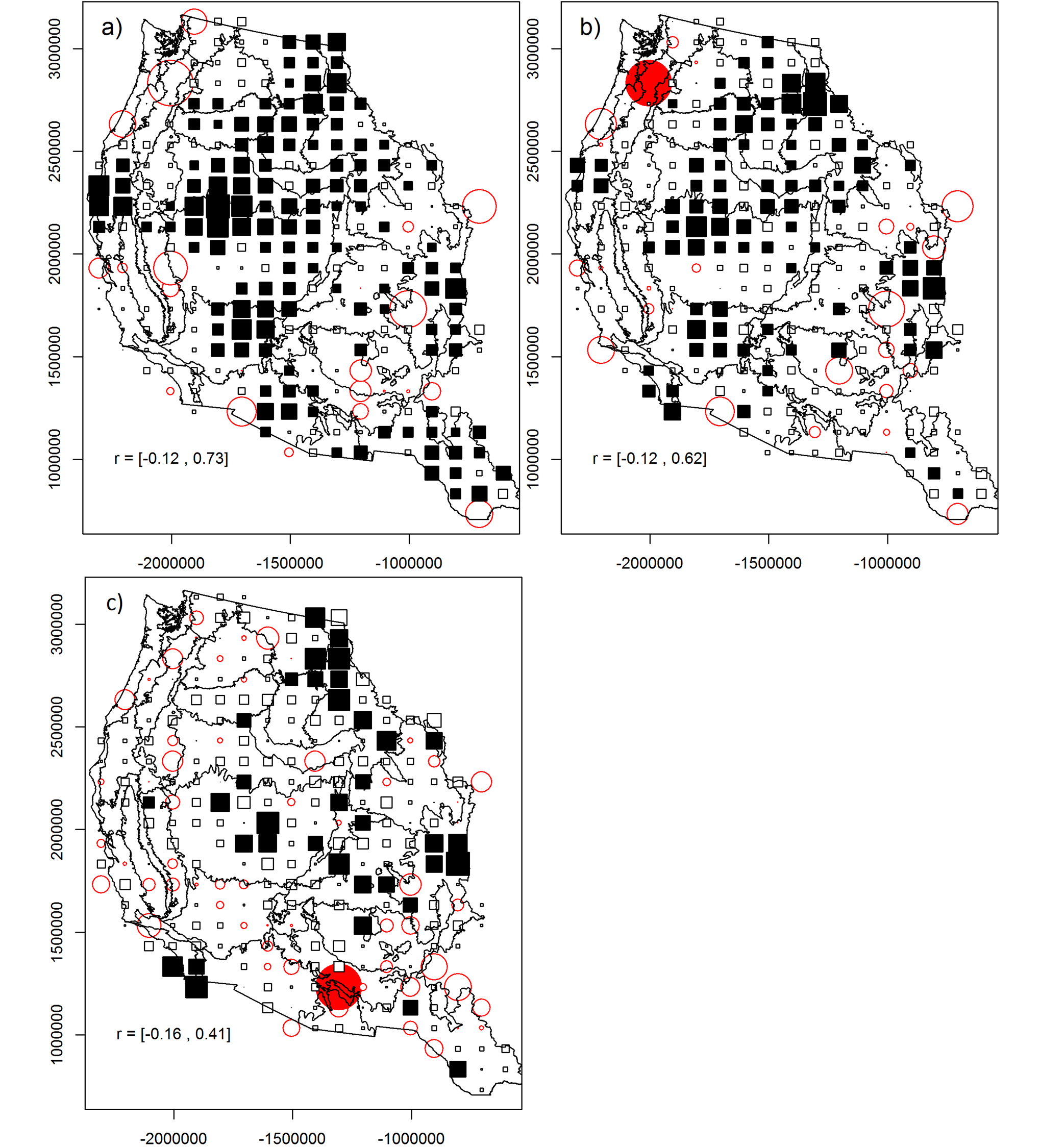

Supplement: S4 Fig — Square and circle symbols indicate positive and negative associations, respectively. Sizes of the symbols indicate strength of association. Filled symbols indicate significant (p < 0.05) associations. (TIF) [file pone.0140839.s004.tif]

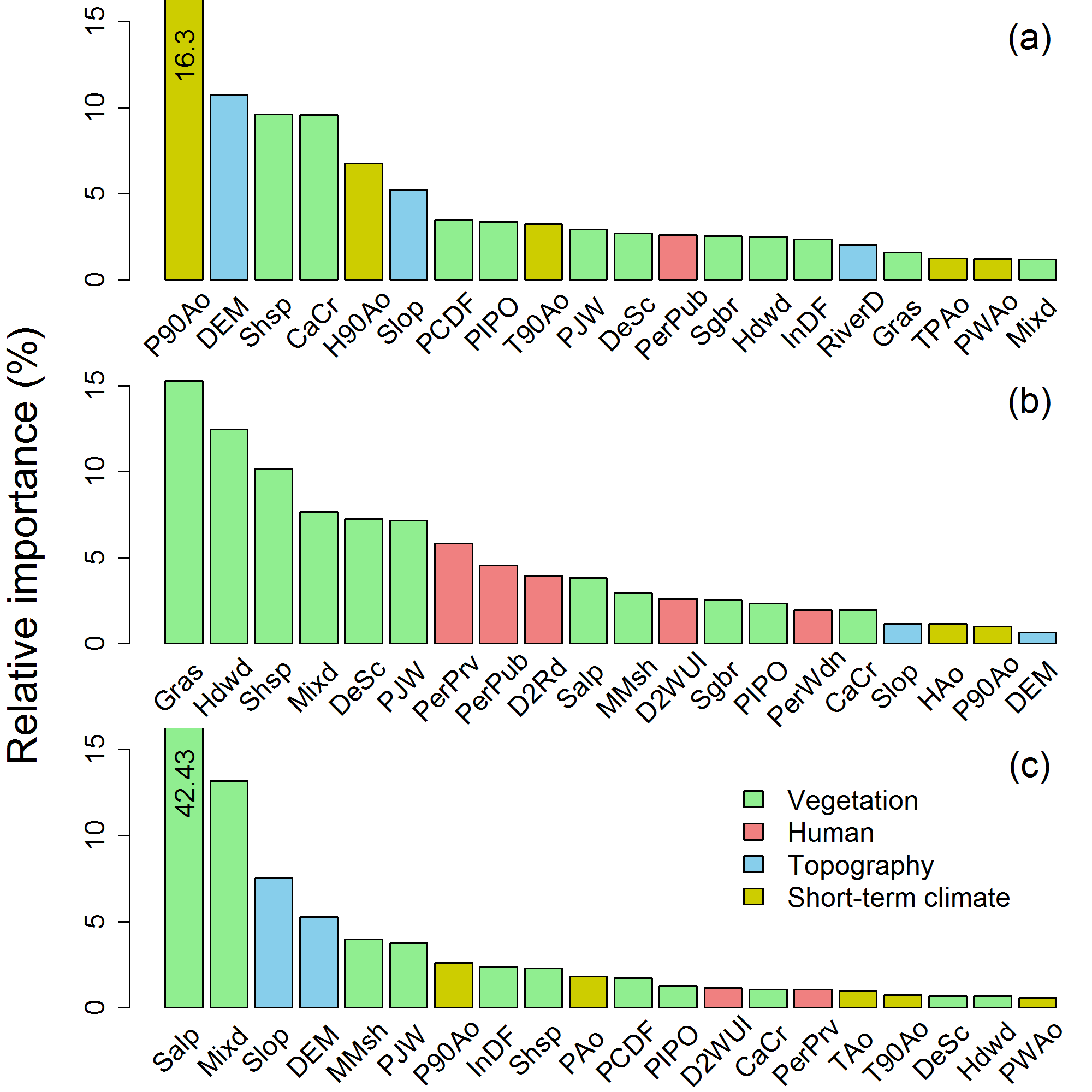

Supplement: S5 Fig — Values are specified for truncated bars. Abbreviations of variables and their corresponding full names are described in Table 1. (TIF) [file pone.0140839.s005.tif]

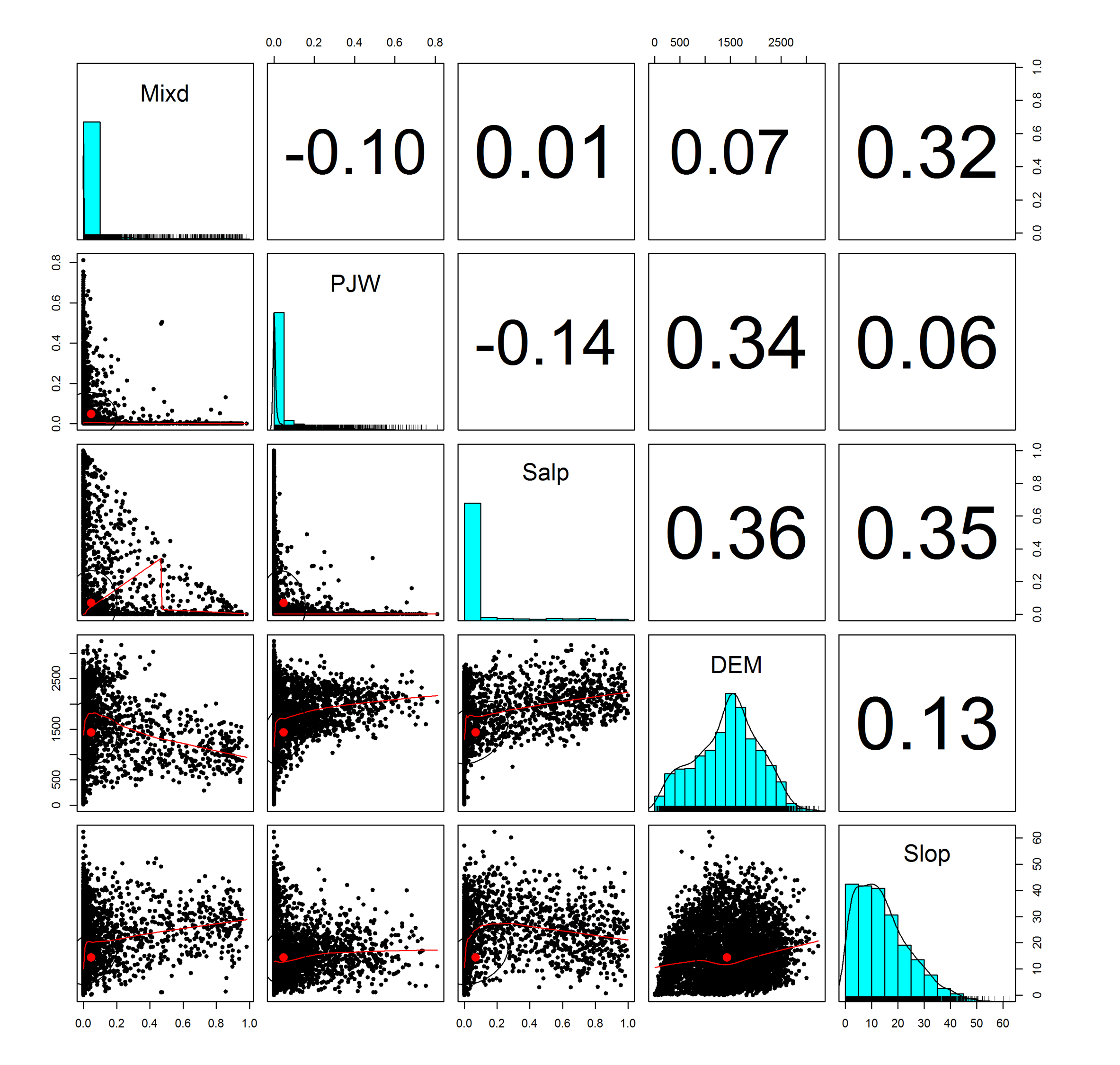

Supplement: S6 Fig — Abbreviations of variables and their corresponding full names are described in Table 1. (TIF) [file pone.0140839.s006.tif]

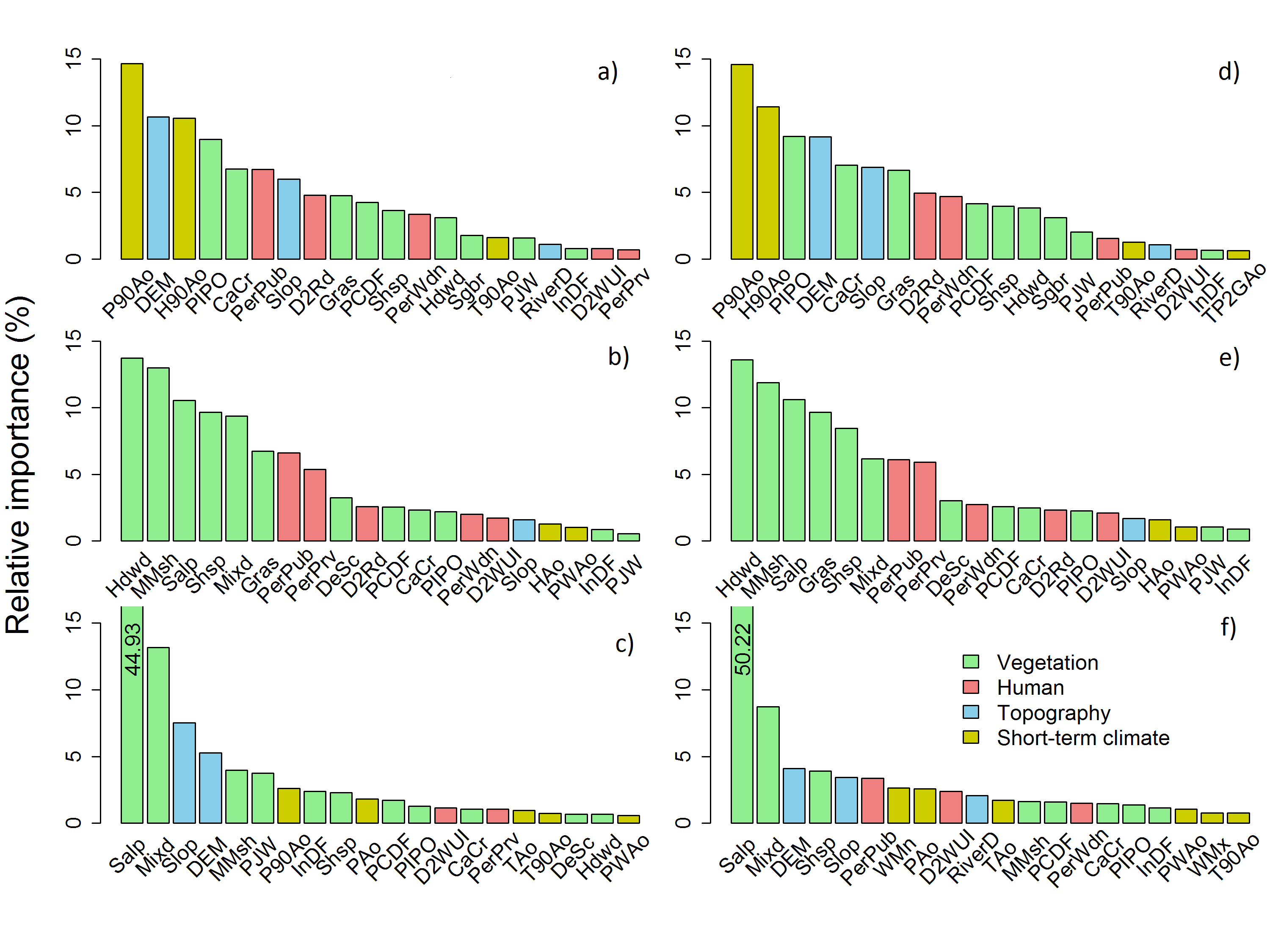

Supplement: S7 Fig — Values are specified for truncated bars. Abbreviations of variables and their corresponding full names are described in Table 1. (TIF) [file pone.0140839.s007.tif]
