# Supplementary material for: Climatic and Landscape Influences on Fire Regimes from 1984 to 2010 in the Western United States
Source: PLoS One. 2015 Oct 14;10(10):e0140839. doi: 10.1371/journal.pone.0140839 (PMC4605733; doi:10.1371/journal.pone.0140839)
Supplement: S1 File — (DOCX) [file pone.0140839.s008.docx]

**S1 File. Data source and processing procedure for fire variables**

## *Fire dataset*

Fire dataset was obtained from MTBS data. We only used large wildfires (>=405 ha) from 1984-2010 for analysis. Although some smaller fires were also included in the MTBS data, the coverage was not complete and we therefore excluded fires less than 405 ha from our analysis to have a consistent size cutoff. Fire perimeters from MTBS were delineated from on-screen interpretation of 30 m resolution dNBR images.

Fire severity was assessed by the degree of change in vegetation (i.e., mortality, or biomass consumption) one year post-fire relative to pre-fire conditions, as measured using spectral indices (e.g., dNBR, RdNBR) derived from Landsat imagery [[1](#_ENREF_1)]. dNBR and RdNBR were two burn severity metrics used widely around the world. Despite their widespread use, they also have significant limitations including inadequate characterization of fire effects on the surface and ground layers, reduced sensitivity at higher NBR values, and inconsistent relationships with field-based measurements across different fires and vegetation types [[2](#_ENREF_2)]. High burn severity was classified by the MTBS project using a custom threshold for each fire using based on the remote sensing indices, plot data, expert knowledge, and published literature [[1](#_ENREF_1)]. Given the subjectivity of this procedure, we focused on the high severity class (> 80% overstory vegetation mortality), which causes the most distinctive change in spectral signatures and therefore should be more consistently classified than lower burn severity classes. High severity was one of six severity classes in MTBS dataset. More information on pre-processing and specific information about burn severity classification is provided in Eidenshink et al (2007).

*Size* of each fire was computed from fire patch, and was compared with reported size. The regression between patch-derived size and report-derived size indicated no difference between two size data (patch-derived size = 1.064* report-derived size, p<0.001, n = 6071), suggesting fire patch accurately capture the perimeters of burned area.

Burn severity, defined as the degree of change in vegetation (e.g., mortality, or biomass consumption) one year post-fire relative to pre-fire conditions [[3](#_ENREF_3)], was measured by relative differenced normalized burn ratio (RdNBR; [[4](#_ENREF_4)]) in MTBS data. RdNBR use light reflected from earth in near-infrared (Landsat band 4) and mid-infrared (Landsat band 7) wavelengths to capture fire induced changes in vegetation and soil characteristics [[5](#_ENREF_5)]. RdNBR accounts for heterogeneity of pre-fire vegetation, and provides a more consistent measure of fire severity when evaluating multiple fires across broad regions and diverse vegetation types [[4](#_ENREF_4)]. Based on remote sensing indices, analyst’s expert knowledge and published literature, burn severity were classified into 6 classes: unburned area, unchanged or low severity, moderate severity, high severity, enhanced greenness, and mask area such as clouds, shadows, large water bodies, or other features on the landscape that erroneously affect the severity classification. We overlaid burned severity dataset with each individual fire patch and extracted area of 6 burned severity classes. The area of 6 burned severity classes was compared with reported size, and indicated no difference between two size data (severity-derived size = 1.029* report-derived size, p<0.001, n = 6071), suggesting our approach accurately extracted burned severity data within each fire. Area burned by high severity within one fire perimeter was referred to high severity burned area in this analysis.

*Percent of high severity burning* was calculated as proportion of high severity burned area within each fire patch, with higher value means more area were burned by high severity fires. Percent of high severity burning for each fire can be used an indicator for the severity of fire at patch level, although severity was usually used as a site or stand level index. Comparison of percent of high severity can be used to indicate whether fire was more intense or not.

REFERENCES

1. Eidenshink J, Schwind B, Brewer K, Zhu Z-L, Quayle B, et al. (2007) A project for monitoring trends in burn severity. Fire Ecology 3: 1-19.

2. French NH, Kasischke ES, Hall RJ, Murphy KA, Verbyla DL, et al. (2008) Using Landsat data to assess fire and burn severity in the North American boreal forest region: an overview and summary of results. International Journal of Wildland Fire 17: 443-462.

3. Lentile LB, Holden ZA, Smith AMS, Falkowski MJ, Hudak AT, et al. (2006) Remote sensing techniques to assess active fire characteristics and post-fire effects. International Journal of Wildland Fire 15: 319-345.

4. Miller JD, Knapp EE, Key CH, Skinner CN, Isbell CJ, et al. (2009) Calibration and validation of the relative differenced Normalized Burn Ratio (RdNBR) to three measures of fire severity in the Sierra Nevada and Klamath Mountains, California, USA. Remote Sensing of Environment 113: 645-656.

5. Miller JD, Thode AE (2007) Quantifying burn severity in a heterogeneous landscape with a relative version of the delta Normalized Burn Ratio (dNBR). Remote Sensing of Environment 109: 66-80.
